# Supplementary material for: Role of Flavonoids in the Prevention of AhR-Dependent Resistance During Treatment with BRAF Inhibitors
Source: Int J Mol Sci. 2020 Jul 16;21(14):5025. doi: 10.3390/ijms21145025 (PMC7404066; doi:10.3390/ijms21145025)
Supplement: Supplementary file 1 [file ijms-21-05025-s001.zip › Table S1.pdf]

Flavonoids	Class	Dietary source	References on melanoma	Role in pigmentation	Role in melanoma	Regulation of AhR activity
**Apigenin**	Flavones	parsley, celery, celeriac, and chamomile	48	Up (27)	Inhibition of proliferation and invasion and induction of apoptosis (1), suppression of PDL1 expression (2), inhibition of STAT3 (3)	Antagonist (4,5)
**(-)-Epigallocatechin Gallate**	Flavan-3-ol	tea	66	Dn (28)	Inhibition of proliferation and invasion by targeting YB1 (6), increase Sorafenib effect (7)	
**Chrysin**	5,7-dihydroxyflavone	honey, propolis, the passion flowers, Passiflora	15	Up (27) or Dn (29)	Synergical effect with IFNa on metastatic pulmonary melanoma (8)	Agonist (9)
**Curcumin**	Curcuminoid	ginger	158	Dn (30)	Inhibition of proliferation Wnt/beta Catenin pathway (10)	Antagonist (11)
**Diosmin**	Flavone	citrus family rutaceae	3			No effect on TCDD induction (4)
**Fisetin**	Flavonol	strawberries, apples, persimmons, onions and cucumbers	20	Up (27)	Inhibition of migration and invasion by inhibiting HGF/cMET (12)	Antagonist (13)
**Genistein**	Isoflavone	dyer's broom	80	Up (31)		Antagonist for TCDD induction (14)
**Kaempferol**	Flavonol	broccoli, brussels sprouts…	32	Up (27)	Inhibition of proliferation and invasion (15) an tumor growth (16)	Agonist (9)
**Myricetin**	Flavonol	fruits, nuts, berries, tea and in red wine	7	Dn (32)	Inhibition of proliferation and invasion by targeting FAK/paxillin and MAPK pathways (17)	Antagonist (4)
**Naringenin**	Flavanone	grapefruit, orange	19	Up (33)	Inhibition olf proliferation by targeting TRAF6 (18), induction of autophagy (19)	Inhibitor of AhR via HSP90 (20)
**Naringin**	Flavanone	cirtrus fruit	6	Dn (34)		
**Nobiletin**	Flavone	citrus and tangerine peel	5	Dn (35)	Inhibition of proliferation (21) by targeting MEK1/2 and RSK2 (22)	
**Puerarin**	Isoflavone	pueraria, kuzdu	0	Up (36)		
**Quercetin**	Flavonol	red onions and kale	120	Dn (37)	Inhibitor of glucose metabolism of melanoma cells by targeting c-SRC (23)	
**Resveratrol**	Stilbenoid	grapes, blueberries, raspberries, mulberries, and peanuts	111	Dn (38)	Inhibition olf proliferation by inducing apoptosis (25), increase of BRAFi sensitivity (26)	Antagonist (26)
**Rutin**	Flavonol	cirtrus fruit	26	Dn (34)		Antagonist (24)
**Silibinin**	Flavonolignans	Silybum marianum	14			
**Tangeretin**	Flavone	citrus and tangerine peel	5	Dn (39)		

Supplemented references

1. Zhao, G., Han, X., Cheng, W., Ni, J., Zhang, Y., Lin, J., S. Apigenin inhibits proliferation and invasion, and induces apoptosis and cell cycle arrest in human melanoma cells. *Oncol. Rep.* **37**, 2277–2285 (2017).
2. Xu, L. *et al.* Apigenin suppresses PD-L1 expression in melanoma and host dendritic cells to elicit synergistic therapeutic effects. *J. Exp. Clin. Cancer Res.* **37**, 261 (2018).
3. Cao, H. H. *et al.* Inhibition of the STAT3 signaling pathway contributes to apigenin-mediated anti-metastatic effect in melanoma. *Sci. Rep.* **6**, 1–12 (2016).
4. Zhang, S., Qin, C. & Safe, S. H. Flavonoids as aryl hydrocarbon receptor agonists/antagonists: Effects of structure and cell context. *Environ. Health Perspect.* **111**, 1877–1882 (2003).
5. Jin, U.-H. *et al.* Structure-Dependent Modulation of Aryl Hydrocarbon Receptor-Mediated Activities by Flavonoids. *Toxicol. Sci.* **164**, 205–217 (2018).
6. Sechi, M. *et al.* Fisetin targets YB-1/RSK axis independent of its effect on ERK signaling: insights from in vitro and in vivo melanoma models. *Sci. Rep.* **8**, 1–14 (2018).
7. Pal, H. C. *et al.* Fisetin, a dietary flavonoid, augments the anti-invasive and anti-metastatic potential of sorafenib in melanoma. *Oncotarget* **7**, (2016).
8. Álvarez, N., Vicente, V. & Martínez, C. Synergistic Effect of Diosmin and Interferon-α on Metastatic Pulmonary Melanoma. *Cancer Biother. Radiopharm.* **24**, 347–352 (2009).
9. Ciolino, H. P., Wang, T. T. Y. & Yeh, G. C. Diosmin and diosmetin are agonists of the aryl hydrocarbon receptor that differentially affect cytochrome P450 1A1 activity. *Cancer Res.* **58**, 2754–2760 (1998).
10. Srivastava, N. S. & Srivastava, R. A. K. Curcumin and quercetin synergistically inhibit cancer cell proliferation in multiple cancer cells and modulate Wnt/β-catenin signaling and apoptotic pathways in A375 cells. *Phytomedicine* **52**, 117–128 (2019).
11. Fukuda, S., Kawase, M., Yamashita, Y. & Ashida, H. Curcumin and its derivatives inhibit 2,3,7,8,-tetrachloro-dibenzo-p-dioxin-induced expression of drug metabolizing enzymes through aryl hydrocarbon receptor-mediated pathway AU - Nakai, Rika. *Biosci. Biotechnol. Biochem.* **82**, 616–628 (2018).
12. Cao, H. H. *et al.* Quercetin inhibits HGF/c-Met signaling and HGFstimulated melanoma cell migration and invasion. *Mol. Cancer* **14**, 1–12 (2015).
13. Perepechaeva ML, Seredina TA, Sidorova YA, Pivovarova EN, Markel AL, Lyakhovich VV, G. A. Letter to the Editor Quercetin Attenuates Benzo (α) pyrene-induced. *Sci, Biomed Env.* **30**, 308–313 (2017).
14. MacPherson, L. & Matthews, J. Inhibition of aryl hydrocarbon receptor-dependent transcription by resveratrol or kaempferol is independent of estrogen receptor α expression in human breast cancer cells. *Cancer Lett.* **299**, 119–129 (2010).
15. Chen, H.-Y. *et al.* Chrysin inhibit human melanoma A375.S2 cell migration and invasion via affecting MAPK signaling and NF-κB signaling pathway in vitro. *Environ. Toxicol.* **0**, (2018).
16. Sassi, A. *et al.* Chrysin, a natural and biologically active flavonoid suppresses tumor growth of mouse B16F10 melanoma cells: In vitro and In vivo study. *Chem. Biol. Interact.* **283**, 10–19 (2018).
17. Cui, S. *et al.* Genistein inhibits the growth and regulates the migration and invasion abilities of melanoma cells via the FAK/paxillin and MAPK pathways. *Oncotarget* **8**, 21674–21691 (2017).

18. Zhang, J. *et al.* Epigallocatechin-3-gallate(EGCG) suppresses melanoma cell growth and metastasis by targeting TRAF6 activity. *Oncotarget* **7**, 79557–79571 (2016).
19. Xie, J. *et al.* A novel ECG analog 4-(S)-(2,4,6-trimethylthiobenzyl)-epigallocatechin gallate selectively induces apoptosis of B16-F10 melanoma via activation of autophagy and ROS. *Sci. Rep.* **7**, 1–14 (2017).
20. Palermo, C. M., Hernando, J. I. M., Dertinger, S. D., Kende, A. S. & Gasiewicz, T. A. Identification of Potential Aryl Hydrocarbon Receptor Antagonists in Green Tea. *Chem. Res. Toxicol.* **16**, 865–872 (2003).
21. Makhmalzadeh, B. S. *et al.* Functionalized Caprolactone-Polyethylene Glycol Based Thermo-Responsive Hydrogels of Silibinin for the Treatment of Malignant Melanoma. *J. Pharm. Pharm. Sci.* **21**, 143 (2018).
22. Lee, M. H. *et al.* Direct targeting of MEK1/2 and RSK2 by silybin induces cell-cycle arrest and inhibits melanoma cell growth. *Cancer Prev. Res.* **6**, 455–465 (2013).
23. Guo, B., Zhang, Y., Hui, Q., Wang, H. & Tao, K. Naringin suppresses the metabolism of A375 cells by inhibiting the phosphorylation of c-Src. *Tumor Biol.* **37**, 3841–3850 (2016).
24. Hamada, M. *et al.* TCDD-Induced CYP1A1 Expression, an Index of Dioxin Toxicity, Is Suppressed by Flavonoids Permeating the Human Intestinal Caco-2 Cell Monolayers. *J. Agric. Food Chem.* **54**, 8891–8898 (2006).
25. Zhao, H. *et al.* Resveratrol induces apoptosis in human melanoma cell through negatively regulating Erk/PKM2/Bcl-2 axis. *Onco. Targets. Ther.* **11**, 8995–9006 (2018).
26. Corre, S. *et al.* Sustained activation of the Aryl hydrocarbon Receptor (AhR) transcription factor promotes the resistance to BRAF inhibitors in melanoma. *Nat. Commun.* in press (2018).
27. Liu-Smith, F., Meyskens, F., Irvine Hall, A. & Meyskens, F. L. Molecular mechanisms of flavonoids in melanin synthesis and the potential for the prevention and treatment of melanoma HHS Public Access. *J. Mol Nutr Food Res* **60**, 1264–1274 (2016).
28. Kim, Y. C., Choi, S. Y. & Park, E. Y. Anti-melanogenic effects of black, green, and white tea extracts on immortalized melanocytes. *J. Vet. Sci.* **16**, 135–143 (2015).
29. Kim, D.-C., Rho, S.-H., Shin, J.-C., Park, H. H. & Kim, D. Inhibition of melanogenesis by 5,7-dihydroxyflavone (chrysin) via blocking adenylyl cyclase activity. *Biochem. Biophys. Res. Commun.* **411**, 121–125 (2011).
30. Tu, C.-X. *et al.* Curcumin Inhibits Melanogenesis in Human Melanocytes. *Phyther. Res.* **26**, 174–179 (2012).
31. Wang, H.-Z., Zhang, Y., Xie, L.-P., Yu, X.-Y. & Zhang, R.-Q. Effects of genistein and daidzein on the cell growth, cell cycle, and differentiation of human and murine melanoma cells11The abbreviations used are: BSA, bovine serum albumin; FBS, fetal bovine serum; ECM, extracellular matrix; DMSO, dimethyl sulfoxide; PTK, protein tyrosine kinase. *J. Nutr. Biochem.* **13**, 421–426 (2002).
32. Juang, L.-J. *et al.* Safety assessment, biological effects, and mechanisms of Myrica rubra fruit extract for anti-melanogenesis, anti-oxidation, and free radical scavenging abilities on melanoma cells. *J. Cosmet. Dermatol.* **18**, 322–332 (2019).
33. Huang, Y.-C., Yang, C.-H. & Chiou, Y.-L. Citrus flavanone naringenin enhances melanogenesis through the activation of Wnt/β-catenin signalling in mouse melanoma cells. *Phytomedicine* **18**, 1244–1249 (2011).
34. Takekoshi, S., Nagata, H., Kitatani, K., Biology, C. & Defense, H. Flavonoids Enhance Melanogenesis in Human Melanoma Cells. **39**, 116–121 (2014).
35. Kim, H. J., Yonezawa, T., Teruya, T., Woo, J.-T. & Cha, B.-Y. Nobiletin, a Polymethoxy

Flavonoid, Reduced Endothelin-1 Plus SCF-Induced Pigmentation in Human Melanocytes. *Photochem. Photobiol.* **91**, 379–386 (2015).
36. Ding, X. *et al.* Effect of puerarin on melanogenesis in human melanocytes and vitiligo mouse models and the underlying mechanism. *Phyther. Res.* **33**, 205–213 (2019).
37. Fan, M., Zhang, G., Hu, X., Xu, X. & Gong, D. Quercetin as a tyrosinase inhibitor: Inhibitory activity, conformational change and mechanism. *Food Res. Int.* **100**, 226–233 (2017).
38. Newton, R. A., Cook, A. L., Roberts, D. W., Helen Leonard, J. & Sturm, R. A. Post-Transcriptional Regulation of Melanin Biosynthetic Enzymes by cAMP and Resveratrol in Human Melanocytes. *J. Invest. Dermatol.* **127**, 2216–2227 (2007).
39. Yoshizaki, N., Hashizume, R. & Masaki, H. A polymethoxyflavone mixture extracted from orange peels, mainly containing nobiletin, 3,3',4',5,6,7,8-heptamethoxyflavone and tangeretin, suppresses melanogenesis through the acidification of cell organelles, including melanosomes. *J. Dermatol. Sci.* **88**, 78–84 (2017).
