# Supplementary material for: Role of Flavonoids in the Prevention of AhR-Dependent Resistance During Treatment with BRAF Inhibitors
Source: Int J Mol Sci. 2020 Jul 16;21(14):5025. doi: 10.3390/ijms21145025 (PMC7404066; doi:10.3390/ijms21145025)
Supplement: Supplementary file 1 [file ijms-21-05025-s001.zip › Table S2.pdf]

Table 2

Flavonoids	Inhibition of AhR activity	Increase of BRAFi sensitivity	Inhibition of resistance	Inhibition of proliferation	Inhibition of migration	Inhibition of invasion
**Apigenin**	+++	+++	+	++	+++	+++
**Chrysin**	+++	++	+	++	+	++
**Fisetin**	+	++	-	-	+++	-
**Kaempferol**	+	++	+	-	-	-
**Naringenin**	-	-	-	-	+/-	+/-
**Resveratrol**	+	++	-	-	-	-
**Silibinin**	+/-	-				
**CH-223191**	+++	+++	+	-	-	+++
