# Supplementary figures and images for: Role of Flavonoids in the Prevention of AhR-Dependent Resistance During Treatment with BRAF Inhibitors
Source: Int J Mol Sci. 2020 Jul 16;21(14):5025. doi: 10.3390/ijms21145025 (PMC7404066; doi:10.3390/ijms21145025)

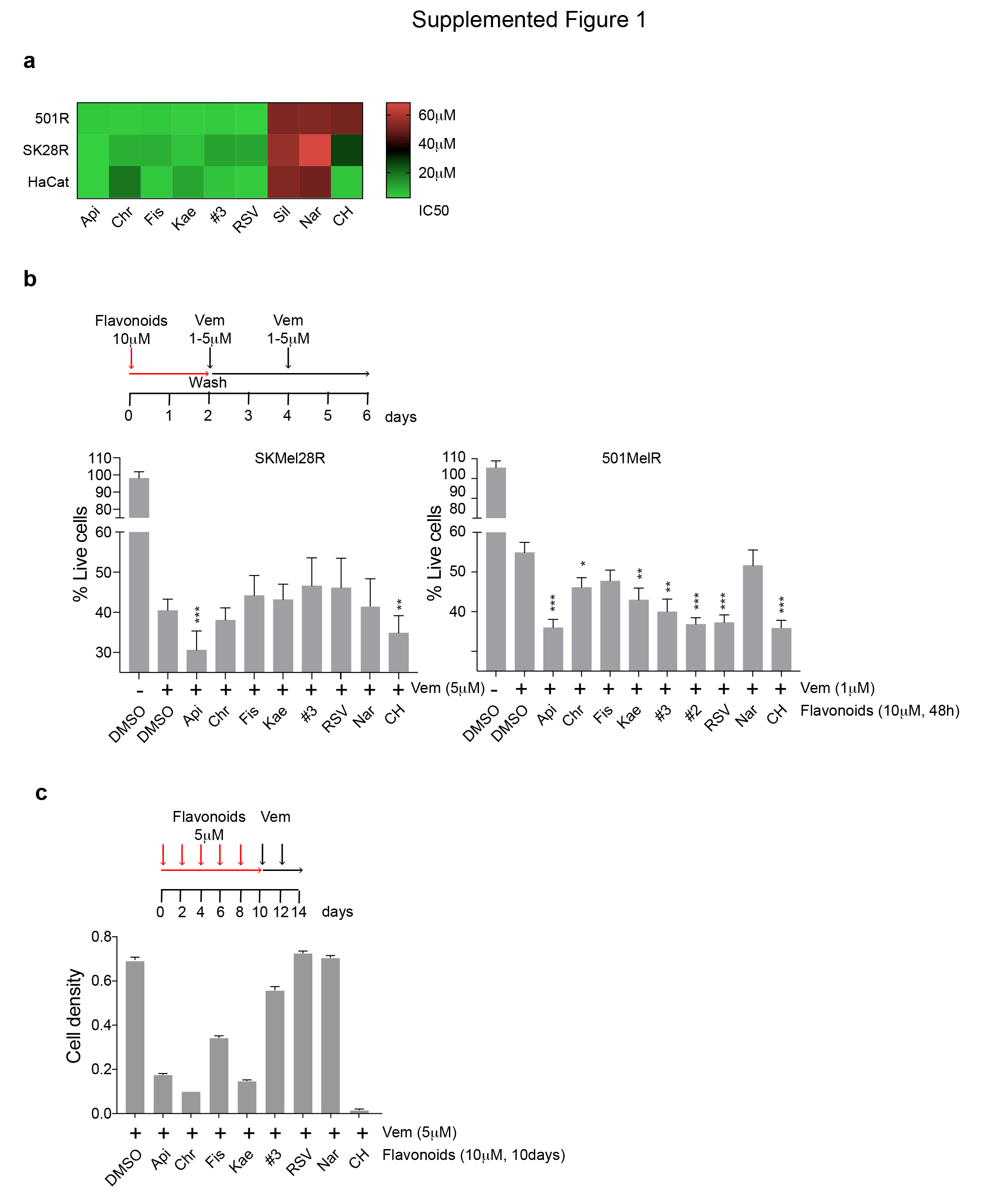

Supplement: Supplementary file 1 [file ijms-21-05025-s001.zip › Figure Sup 1.tif]

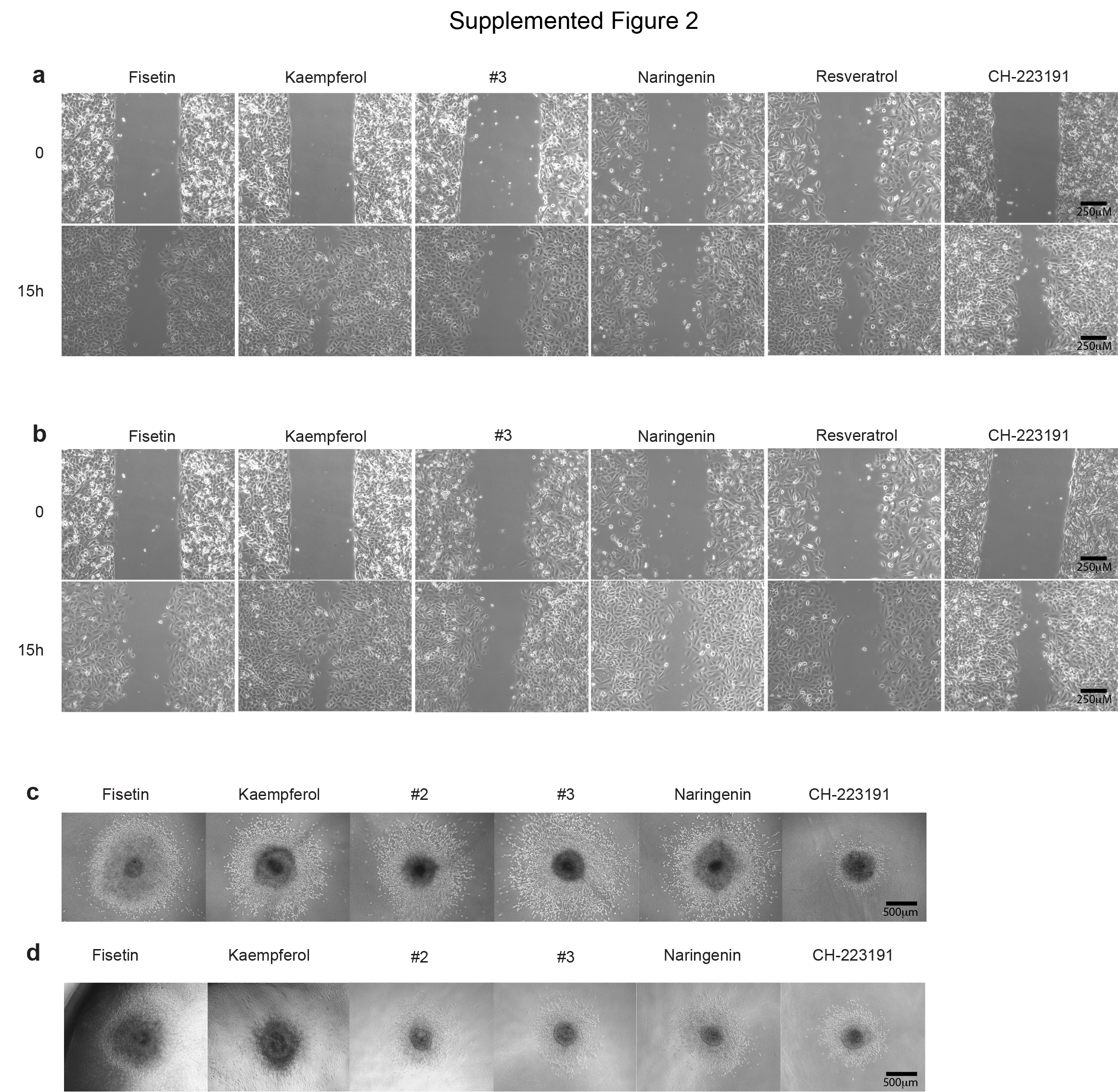

Supplement: Supplementary file 1 [file ijms-21-05025-s001.zip › Figure Sup 2.tif]

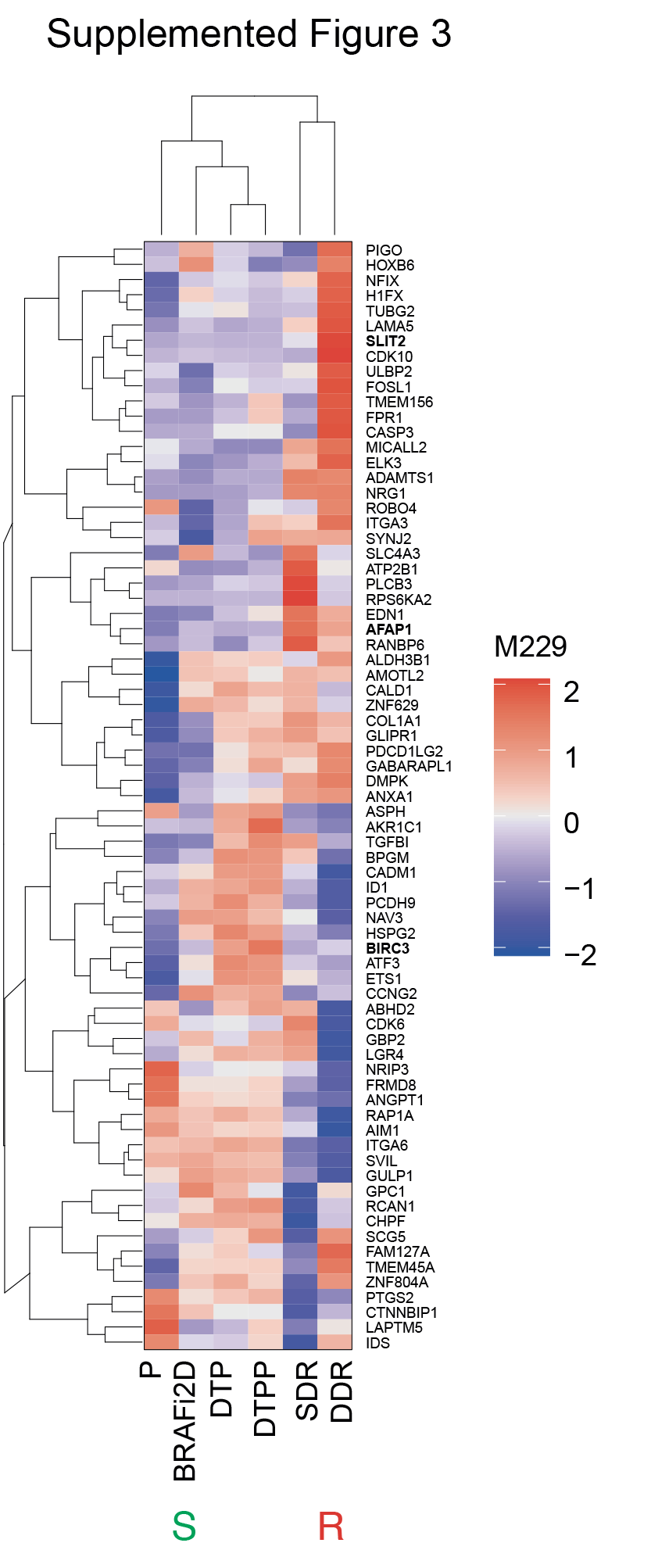

Supplement: Supplementary file 1 [file ijms-21-05025-s001.zip › Figure Sup 3.tif]
